# Topical glyceryl trinitrate to increase radial artery diameter in neonates: study protocol for a randomized controlled trial

**DOI:** 10.62675/2965-2774.20240235-en

**Published:** 2024-03-25

**Authors:** Deepika Wagh, Dinesh Pawale, Sanjay Patole, Shripada Rao

**Affiliations:** 1 Perth Children's Hospital Department of Neonatology Neonatal Clinical Care Unit Nedlands Perth Australia Neonatal Clinical Care Unit, Department of Neonatology, Perth Children's Hospital - Nedlands, Perth, Western Australia.; 2 King Edward Memorial Hospital Department of Neonatology Subiaco Perth Western Australia Department of Neonatology, King Edward Memorial Hospital - Subiaco, Perth, Western Australia.

**Keywords:** Catheterization, peripheral, Radial artery, Nitroglycerin, Infant, newborn, Infant, Intensive care units, neonatal

## Abstract

**Background:**

Newborn infants admitted to the neonatal intensive care unit require arterial cannulation for hemodynamic monitoring and blood sampling. Arterial access is achieved through catheterization of umbilical or peripheral arteries. Peripheral artery cannulation is performed in critically ill newborns, but artery localization and cannulation is often challenging and unsuccessful. Therefore, increasing the internal diameter and preventing vasospasm are important for successful peripheral artery cannulation in neonates. Topical glyceryl trinitrate has the potential to increase cannulation success by relaxing arterial smooth muscles and thus increasing the internal diameter. We aim to conduct a pilot randomized controlled trial to evaluate the efficacy and safety of topycal glyceryl trinitrate in increasing the diameter of the radial artery in neonates.

**Methods/Design:**

This study will be a single-center, observer-blind, randomized, placebo-controlled trial conducted in the neonatal intensive care unit of Perth Children's Hospital, Western Australia. A total of 60 infants born at >34 weeks of gestation who are admitted for elective surgery or medical reasons and for whom a peripheral arterial line is needed for sampling or blood pressure monitoring will be recruited after informed parental consent is obtained. The primary outcome will be the change in radial arterial diameter from baseline to postintervention. Secondary outcomes will be the absolute and percentage change from baseline in the radial arterial diameter in both limbs and safety (hypotension and methemoglobinemia).

**Discussion:**

This will be the first randomized controlled trial evaluating the use of topical glyceryl trinitrate to facilitate peripheral artery cannulation in neonates. If our pilot randomized controlled trial confirms the benefits of glyceryl trinitrate patches, it will pave the way for large multicenter randomized controlled trials in this field.

## INTRODUCTION

Children admitted to intensive care units (ICUs) require arterial cannulation for hemodynamic monitoring and blood sampling. In neonates, arterial access can be achieved through umbilical artery catheterization in the first 3 - 4 days of life, whereas in older infants and children, it is achieved via peripheral arteries. Even in neonates, umbilical arterial catheterization may not be feasible (e.g., in neonates with abdominal surgical conditions and omphalitis). The radial, posterior tibial, and brachial arteries are commonly used for cannulation in children and neonates. Radial artery cannulation is also used for percutaneous cardiac intervention. Radial arteries are of small caliber, which makes their localization and cannulation challenging and potentially unsuccessful. The radial artery is the most frequently used site for cannulation, as it is superficial and has a relatively large diameter compared to other peripheral arteries. Repeated cannulation attempts can lead to bleeding, spasm and dissection of the peripheral arteries.^(1)^ Ultrasound-guided insertion can increase cannulation success but requires high expertise.^(2,3)^ A recent pediatric audit found that even with ultrasound, the first attempt success rate was only 31%.^(4)^ Resource-limited settings may be limited by the cost of ultrasound equipment and maintenance. Therefore, increasing the internal diameter and preventing vasospasm are important for successful peripheral artery cannulation in neonates.

The use of glyceryl trinitrate (GTN) as a local application has the potential to increase cannulation success by increasing the diameter of peripheral arteries and preventing vasospasm. Glyceryl trinitrate is converted to nitric oxide in vascular smooth muscle, which activates guanylate cyclase and increases the level of cyclic guanosine monophosphate. This relaxes arterial smooth muscle, leading to vasodilation.^(5)^ Adult studies have shown that locally applied GTN increases the internal diameter of peripheral arteries, improves the success rate of cannulation, and reduces the risk of vasospasm and occlusions during cardiac catheterization.^(6-8)^

Our recent systematic review^(9)^ found only two studies addressing this issue in the pediatric population. One study used a topical patch of GTN^(10)^, while the other study used subcutaneous GTN.^(11)^ Both studies found that local GTN improved the success rates and shortened the time taken for arterial cannulation. However, the sample size was very small. Moreover, the neonatal population was not represented in these studies.

We hypothesize that the local application of GTN in the form of a transdermal patch would achieve adequate dilation of the radial artery and increase the first-attempt success rate of peripheral artery cannulation in neonates.

We aim to conduct a pilot randomized controlled trial to evaluate the efficacy and safety of local GTN in increasing the diameter of the radial artery in neonates.

## Methods/Design

### Aim and hypothesis

We aim to conduct a randomized controlled trial to establish the effectiveness of local GTN in increasing the diameter of the radial artery in neonates.

We hypothesize that local application of GTN in the form of a transdermal patch will result in adequate dilation (by at least 25% from the baseline diameter) of the radial artery in neonates.

### Objectives

The specific objectives are:

1. To confirm that GTN patch application for 30 - 60 minutes increases the radial artery diameter of neonates.
2. To confirm that it is safe to apply a GTN patch for 30 - 60 minutes for neonates.
3. To estimate the mean values of the within-patient changes in the peripheral artery diameter from baseline to inform the choice of the optimal duration of GTN patch application in the context of cannulation in neonates.
4. To evaluate the feasibility of a proposed future trial design to assess the impact of GTN patches on first-attempt cannulation rates in terms of recruitment and data collection.

#### Trial hypothesis being tested:

The topical application of GTN for 30 - 60 minutes increases the radial artery diameter compared to no application.

### Study design, setting and time period

This prospective, parallel, single-center randomized controlled trial will be conducted in the neonatal ICU of Perth Children's Hospital (PCH), Child and Adolescent Health Service (CAHS), Perth, Australia. The PCH neonatal ICU is a 30-bed tertiary referral center that provides intensive and high dependency care for critically ill neonates. Over 800 infants are admitted to the PCH neonatal ICU each year, and all infants are born at other hospitals. This study has been approved by the CAHS Human Research Ethics (HREC) and Governance Committees and is on the Australian New Zealand Clinical Trials Registry. The study will aim to recruit eligible patients (n = 60) from July 2023 to July 2024. Newborns’ parents or guardians will be approached, and written informed consent will be obtained. The assessment plan is shown in table 1. This clinical protocol has been prepared according to the SPIRIT 2013 Checklist.^(12)^

**Table 1:** Assessment plan

Informed consent
Inclusion/exclusion criteria review
Randomization
Measurement of the radial artery diameter by ultrasound preintervention by a blinded operator
Application of the GTN patch/bandage with no patch by unblinded nursing staff (not involved in the day-to-day management of the baby) for 30 minutes or 45 minutes or 60 minutes
Blood pressure measurement performed by the bedside nurse every 15 minutes until the end of the intervention
Tracking of oxygen saturation and FiO_2_ requirements by the bedside nurse
Blood gas measurement by the bedside nurse postintervention to measure methemoglobin levels
Removal of the GTN patch/bandage with no patch by unblinded nursing staff (not involved in the day-to-day management of the baby) for 30 minutes or 45 minutes or 60 minutes
Measurement of the radial artery diameter by ultrasound postintervention by a blinded operator

GTN - glyceryl trinitrate; FiO_2_ - fraction of inspired oxygen.

### Inclusion criteria

1. Neonates born at > 34 weeks of gestation who are admitted to the neonatal ICU at PCH and for whom a peripheral arterial line is needed for sampling or invasive blood pressure monitoring.
2. Neonates born at > 34 weeks of gestation admitted to the neonatal ICU, scheduled to undergo elective or general surgery and for whom peripheral artery cannulation is needed for hemodynamic monitoring or frequent blood sampling (perioperative).

### Exclusion criteria

1. Neonates who undergo previous radial artery cannulation attempts and those who have hematomas at the cannulation site, abnormal Allen's test results, a hypercoagulable state, coagulopathy, and peripheral vascular disease.
2. Neonates with unstable vital signs, including hypotension, shock or significant arrythmias.
3. Neonates scheduled to undergo cardiac surgery.
4. Neonates with increased intracranial pressure, intracranial hemorrhage, and recent use of sildenafil.
5. Neonates with a visible deformity in the radial artery area.

### Interventions

Glyceryl trinitrate 5mg (Transiderm-Nitro patch, 5mg/24 hours) will be applied for 30 minutes, 45 minutes or 60 minutes. Neonates will be allocated to either the GTN Group or the Control Group. Glyceryl trinitrate is a registered medication in the Australian Therapeutic Goods Registry. The Therapeutic Goods Administration (TGA) has been notified about this clinical trial (Reference number: CT-2023-CTN-02133-1).

GTN Group: for neonates in this group, a GTN patch (5mg) will be applied at the site of radial artery cannulation for 30 minutes (Group A, n = 15), 45 minutes (Group B, n = 15) or 60 minutes (Group C, n = 15). Immediately after its application, the GTN patch will be covered with a Coban™ adhesive bandage to ensure blinding.

Control Group: for neonates in this group (Group D, n = 15), a Coban™ adhesive bandage with no intervention will be applied to the bare skin at the site of the radial artery for 30 minutes (n = 5), 45 minutes (n = 5) or 60 minutes (n = 5) to ensure blinding.

Only one arterial site per infant will be studied to ensure that the samples are independent.

### Justification of the dose of the intervention

Patients randomized to the active arms (Groups A, B and C) will have a GTN patch 5mg (Transiderm-Nitro patch, 5mg/24 hours) applied for 30 minutes, 45 minutes or 60 minutes, and a Coban™ adhesive bandage will be placed over the patch. Minitran 5 has a surface area of 6.7cm^2^ and the amount of glyceryl trinitrate (GTN) released over 24 hours is 5mg. The Transiderm-Nitro transdermal delivery system is designed to provide continuous controlled release of GTN through intact skin to overcome the problems of the short half-life and extensive first-pass metabolism of GTN. The rate of release of GTN is linearly dependent upon the area of the applied system.

The release rate from the patch is 0.2mg/hour. The onset of action of the GTN patch occurs within 30 minutes. It has been shown that GTN levels rise to a steady state within 1 - 2 hours of application of the GTN patch, and the levels wane with a half-life of approximately 30 min after removal of the patch.^(13)^

The studies performed by Hasanin et al. (GTN transdermal patch)^(10)^ and Jang et al. (subcutaneous injection of GTN) were used for pharmacokinetic data and dose selection.^(11)^ Both of these studies used a dose of 2.5 - 5mg/kg/hour. Most of the available data for GTN dosage were derived from adult, not pediatric, studies.^(7,14,15)^ There were no major systemic side effects, such as hypotension and methemoglobinemia, reported in the pediatric studies.^(9)^

### Study endpoint

The primary endpoint of the study is radial artery diameter. After obtaining informed consent from a patient's parent, images of the radial artery will be obtained from the infant's wrist using a portable ultrasound machine. The diameter of the radial artery will be measured using a linear, high-frequency hockey stick probe (6 - 113MHz) with the Philips EPIQ CVx Diagnostic Ultrasound system.

Measurements will be obtained in the short-axis plane of the distal forearm, medial to the border of the styloid process of the radius and at the point with maximal pulsation of the radial artery, with the wrist at a 45º angle.^(16)^ The point will be marked to guide the intervention and ensure coverage of the site over the peripheral artery, which will be measured postintervention. The operator will be an experienced neonatologist blinded to the study group. Measurements will be obtained in both radial arteries at baseline before application of the GTN patch/bandage with no patch and at 30 minutes, 45 minutes or 60 minutes after application.

### Participant withdrawal criteria

The mean and systolic blood pressure will be recorded every 15 minutes in both the GTN and Control Groups until the intervention ends. If any participant in either the GTN Group or Control Group develops hypotension (defined as a > 30% drop in systolic blood pressure from baseline) and/or any sudden increase in the oxygen requirement with an increase in the methemoglobin levels (determined by blood gas analysis), they will be removed from the study. Participants can withdraw from the study at any point.

### Outcomes

#### Primary outcome:

the primary outcome will be the change in the postintervention radial artery diameter from baseline. This will be compared between the GTN and Placebo Groups.

#### Secondary outcomes:

the secondary outcomes will be the postintervention cross-sectional radial artery diameter in the ipsilateral limb and the percentage change in the radial arterial diameter in the ipsilateral limb from baseline. Postintervention absolute and percentage changes in the radial arterial diameter in the contralateral limb from baseline will also be assessed.

#### Safety outcomes:

in this study, the side effects of topical GTN, such as hypotension and methemoglobinemia, will be assessed.

### Participant timeline

The study schedule is shown in table 1.

Duration of participant participation: 30 minutes to 1 hour.

### Sample size

We aim to recruit 60 patients (15 in each arm: 1 control and 3 GTN arms) over 12 months, which is achievable given that we expect approximately 250 eligible patients to be admitted to the neonatal ICU each year.

Justification of the sample size is based on the desire to demonstrate that reasonable vasodilation with GTN patch application is achievable in our target study population. We assume that the measures of the radial artery diameter in study participants will have a mean of 0.76mm without treatment and a common within-group standard deviation of approximately 0.18mm, as observed in a reference study of neonates aged 0 - 6 months.^(12)^ With a sample size of 15 for each group (1 control and 3 GTN patch groups), the margin of error around the corresponding estimated population mean diameters is less than 1 mm, and an omnibus one-way ANOVA would have more than 80% power to determine whether GTN patch application increases the radial artery diameter when the minimum mean of the treatment group differs from that of the control group by approximately 0.19mm (alpha = 0.05). Analyses based on the change from baseline, which is our primary outcome, are expected to be more powerful than analyses assessing the cross-sectional diameters, and the detectable cross-sectional difference of 0.19mm corresponds to an approximate 25% increase from the estimated baseline value, which is of the order previously observed in children aged 0 - 2 years receiving subcutaneous injections of GTN,^(11)^ and substantially less than that observed after patch application in children aged 2 - 8 years (average increase of 40% in the radial artery diameter after 30 minutes and an increase of 70% after 1 hour).^(10)^ Furthermore, power will be increased by extending the assessment of GTN-induced vasodilation to an ANCOVA that reduces residual variation by adjusting for key demographic/clinical correlates of arterial diameter.

### Recruitment

A parent/guardian of an eligible infant will be approached by the coordinating principal investigator (CPI) or the CPI delegate and invited to participate in this study. Informed parental consent for trial participation will be required before elective surgical procedures are performed so that the trial intervention and data collection can be initiated.

## Assignment of interventions

### Random sequence generation

Group assignment will be allocated by the Clinical Trial Pharmacist at PCH using a computer-generated randomization sequence to minimize selection bias.

### Allocation concealment

Concealment will be obtained using opaque sealed envelopes. Once consent is obtained, a nursing staff member will open the next opaque sealed envelope to allocate the infant to the control or intervention group.

### Blinding

Blinding of families, health care providers and study investigators to the group allocation will be performed.

#### Unblinded staff:

a nursing staff member who is not involved in the day-to-day management of the neonate will be responsible for opening the envelope, determining the group assignment and applying the GTN patch/placebo and removing the GTN patch/placebo with no additional participation in the study.

#### Blinded staff:

the ultrasound measurements of the radial artery will be performed by an operator who will be blinded to the intervention. In addition, the bedside nurse who measures the blood pressure every 15 minutes until the end of the intervention will also be blinded.

There will be a blinded and unblinded delegation log for blinded and unblinded trial members with their duties listed.

## Data collection, management, and analysis

### Data collection methods

All clinical data, such as patient characteristics, including gestational age, chronological age, sex, weight, location, main diagnosis, vital signs, medications, and ventilation use, will be collected (Table 2).

**Table 2:** Patient and clinical characteristics

		GTN Group n = 45	Control Group n = 15
Birth weight			
Gestational age			
Date of birth			
Sex			
	Male		
	Female		
Age at intervention			
Diagnoses			
	Perioperative		
	Medical		
Medications			
	None		
	Antibiotics		

GTN - glyceryl trinitrate.

After obtaining informed consent from each patient's parent, images of the radial artery will be obtained from the infant's wrist using a portable ultrasound machine. The diameter of the radial artery will be measured using a linear, high-frequency hockey stick probe (6 - 13MHz) with the Philips EPIQ CVx Diagnostic Ultrasound system.

The operator will be an experienced neonatologist (member of the Australian Society for Ultrasound in Medicine and routinely performing ultrasounds in the unit) who is blinded to the study group. Measurements will be obtained in the radial arteries of both limbs at baseline before application of the GTN patch/bandage with no patch and at 30 minutes, 45 minutes or 60 minutes after application.

### Data management

The trial involves the collection of clinical data and radial artery images by ultrasound measurement. They will be stored in a password-protected database on the hospital computer. CAHS health department information technology will be involved to help secure these data in a password protected system. Sharing or reuse of data will not be performed. Records will be stored in a locked filing cabinet in a secured office in Neonates at Perth Children's Hospital. The study data will be retained per the Western Australia health guidelines. Data collected will be accessed only by the authorized members of the study team named on the HREC application for the study.

### Statistical methods

Table 3 gives a description of the timing and method of assessment of the efficacy and safety parameters. The ultrasound operator and examiner will be blinded to the study.

**Table 3:** Timing and method of assessment of the efficacy and safety parameters

		Measured by	Analysis
Primary efficacy outcome			
	Change in postintervention ipsilateral radial arterial diameter from baseline	Ultrasound at the site of cannulation measured at time of randomization of glyceryl trinitrate/placebo patch and 30, 45 or 60 minutes postintervention	ANCOVA
Secondary outcomes			
	Cross-sectional radial artery diameter in the ipsilateral limb	Measured postintervention by ultrasound	Linear regression
	Percentage change from baseline in radial arterial diameter in ipsilateral limb	Ratio of the postintervention to baseline radial arterial diameters as measured by ultrasound	Linear regression
	Change in the radial artery diameter in the contralateral (no intervention) limb from baseline	Difference and ratio of the radial artery diameter measured at baseline and postintervention	Mixed effects regression modeling of radial artery diameters in both limbs.
Safety outcomes			
	Blood pressure	Systolic blood pressure monitored every 15 minutes	Mixed effects regression modeling of patient trajectories
	Methemoglobinemia	Monitoring of the oxygen requirement and a blood gas analysis if the oxygen requirement increases	Mixed effects regression modeling of patient trajectories

ANCOVA - Analysis of covariance.

### Data and safety monitoring

An independent Data and Safety Monitoring Committee (DSMC) has been established to ensure the safety of the trial participants and to monitor the quality of the study and data collection. The DSMC has a neonatologist and senior pharmacist who are external to PCH and will not be involved in the trial. The DSMC will review the study data and conduct interim analyses following the recruitment of 20 and 40 participants for the key safety outcomes: the incidence of hypotension (defined as a decrease in systolic blood pressure by 30% from the baseline reading or more) and methemoglobinemia (as measured by blood gas analysis if any infant develops a sudden increase in their oxygen requirement after the intervention). The DSMC charter is enclosed as a supplemental file.

All adverse events will be reported to the HREC of CAHS and DSMC within one business day of the first report of the event.

### Ethics and dissemination

Ethics approval for all aspects of this study has been granted (RGS 0000005763). Deviation from this protocol will occur only with prior approval from HREC. The study will be conducted in accordance with the International Council on Harmonization Good Clinical Practice guidance documents and the National Health and Medical Research Council National Statement of ethical conduct in Human Research.

The proposed intervention is noninvasive, and this will be the first study evaluating the use of topical GTN to facilitate peripheral artery cannulation in neonates. The GTN patch is being used in neonates to prevent vasospasm associated with local ischemia after peripheral artery cannulation (in both preterm and term infants). Many neonatal units worldwide use GTN for this purpose. Hence, there are no ethical implications for the newborns assigned to the intervention group.

As neonates are unable to provide consent, informed consent will be obtained prospectively from the parents/guardians of eligible infants for the participation of their newborn in the study. All efforts will be made to maintain the highest standards of care while obtaining consent for clinical research.

The CPI or a delegate will provide the parents/guardians with a trial information sheet. Trial information will be discussed in detail with the parents/guardians, and their questions will be answered in full. Once the parents/caregivers are happy with the information and agree to enrollment, they will sign the HREC-approved consent form prior to randomization.

Participation in this study is voluntary. A parent/guardian can withdraw their infant at any time without giving a reason. No additional information will be collected. We will use the data and radial artery images already collected unless the parent/guardian advises us not to. The withdrawal of participation will not prejudice current or future medical treatments.

The original consent form will be filed in the study data file, and the parents/guardians will be given a copy; documentation about the type of study the infant is enrolled in will be provided.

## DISCUSSION

Given the importance of having reliable arterial access in neonates and the difficulty in cannulation, any intervention that improves the chances of successful peripheral arterial catheterization will help improve the clinical outcomes of critically ill neonates. This research project will help determine if the topical use of GTN for peripheral artery cannulation is beneficial and whether its benefits outweigh the potential side effects. This will be the first study evaluating the use of topical glyceryl trinitrate to facilitate peripheral artery cannulation in neonates. If our pilot RCT confirms the benefits of the GTN patch, it will pave the way for large multicenter RCTs evaluating its efficacy in improving the first-attempt success rate of peripheral artery cannulation in neonates.
