# Supplementary material for: Topical glyceryl trinitrate to increase radial artery diameter in neonates: study protocol for a randomized controlled trial
Source: Crit Care Sci. 2024 Mar 25;36:e20240235en. doi: 10.62675/2965-2774.20240235-en (PMC11098072; doi:10.62675/2965-2774.20240235-en)
Supplement: Translation

# Translation: Trinitrato de glicerilo tópico para aumentar o diâmetro da artéria radial em recém-nascidos: protocolo de estudo de um ensaio clínico randomizado

**DOI:** 10.62675/2965-2774.20240235-pt

**Authors:** Deepika Wagh, Dinesh Pawale, Sanjay Patole, Shripada Rao

**Affiliations:** 1 Nedlands Perth Austrália Ocidental Unidade de Cuidados Clínicos Neonatal, Departamento de Neonatologia, Perth Children's Hospital - Nedlands, Perth, Austrália Ocidental.; 2 Subiaco Perth Austrália Ocidental Departamento de Neonatologia, King Edward Memorial Hospital - Subiaco, Perth, Austrália Ocidental.

**Keywords:** Cateterismo periférico, Artéria radial, Nitroglicerina, Recém-nascido, Lactente, Unidades de terapia intensiva neonatal

## Abstract

**Histórico:**

Os recém-nascidos internados na unidade de terapia intensiva neonatal necessitam de canulação arterial para monitoramento hemodinâmico e coleta de sangue. O acesso arterial é obtido por meio de cateterização das artérias umbilicais ou periféricas. A canulação da artéria periférica é realizada em recém-nascidos em estado grave, mas a localização e a canulação da artéria são, muitas vezes, desafiadoras e sem sucesso. Assim, o aumento do diâmetro interno e a prevenção do vasoespasmo são importantes para o sucesso da canulação da artéria periférica em recém-nascidos. O trinitrato de glicerilo tópico tem o potencial de aumentar o sucesso da canulação ao relaxar a musculatura lisa arterial e, dessa forma, aumentar o diâmetro interno. Nosso objetivo é realizar um ensaio piloto controlado e randomizado para avaliar a eficácia e segurança do trinitrato de glicerilo tópico no aumento do diâmetro da artéria radial em recém-nascidos.

**Métodos/Desenho:**

Este estudo será um estudo de centro único, cego para o observador, randomizado, controlado por placebo, conduzido na unidade de terapia intensiva neonatal do *Perth Children's Hospital*, Austrália Ocidental. Serão recrutados 60 bebês nascidos com mais de 34 semanas de gestação, internados para cirurgia eletiva ou por razões médicas e para os quais é necessária a colocação de um acesso arterial periférico para coleta de amostras ou monitoramento da pressão arterial, após a obtenção do consentimento informado dos pais. O desfecho primário será a mudança no diâmetro arterial radial basal e pós-intervenção. Os desfechos secundários serão a alteração absoluta e percentual basal no diâmetro arterial radial em ambos os membros e a segurança (hipotensão e metemoglobinemia).

**Discussão:**

Este será o primeiro estudo controlado e randomizado a avaliar o uso de trinitrato de glicerilo tópico para facilitar a canulação da artéria periférica em recém-nascidos. Se nosso estudo-piloto randomizado e controlado confirmar os benefícios dos adesivos de trinitrato de glicerilo, ele abrirá caminho para grandes estudos multicêntricos randomizados e controlados nesse campo.

## INTRODUÇÃO

Crianças internadas em unidades de terapia intensiva (UTIs) necessitam de canulação arterial para monitoramento hemodinâmico e coleta de sangue. Em recém-nascidos, o acesso arterial pode ser obtido por cateterização da artéria umbilical nos primeiros 3 a 4 dias de vida, enquanto em lactentes e crianças mais velhas, ele é realizado por artérias periféricas. Mesmo em recém-nascidos, o cateterismo arterial umbilical pode não ser viável (por exemplo, em recém-nascidos com condições cirúrgicas abdominais e onfalite). As artérias radial, tibial posterior e braquial são comumente usadas para canulação em crianças e recém-nascidos. A canulação da artéria radial também é usada para intervenção cardíaca percutânea. As artérias radiais são de pequeno calibre, o que torna sua localização e canulação desafiadoras e potencialmente malsucedidas. A artéria radial é o local mais frequentemente usado para canulação, pois é superficial e tem um diâmetro relativamente grande em comparação com outras artérias periféricas. Tentativas repetidas de canulação podem levar a sangramento, espasmo e dissecção das artérias periféricas.^(1)^ A inserção guiada por ultrassom pode aumentar o sucesso da canulação, mas requer alta especialização.^(2,3)^ Uma auditoria pediátrica recente constatou que, mesmo com o ultrassom, a taxa de sucesso na primeira tentativa foi de apenas 31%.^(4)^ Locais com recursos limitados podem ser prejudicados pelo custo do equipamento de ultrassom e da manutenção. Assim, o aumento do diâmetro interno e a prevenção do vasoespasmo são importantes para o sucesso da canulação da artéria periférica em recém-nascidos.

O uso tópico de trinitrato de glicerilo (TNG) tem o potencial de aumentar o sucesso da canulação, aumentando o diâmetro das artérias periféricas e prevenindo o vasoespasmo. O TNG é convertido em óxido nítrico no músculo liso vascular, que ativa a guanilato ciclase e aumenta o nível de monofosfato de guanosina cíclico. Isso relaxa o músculo liso arterial, levando à vasodilatação.^(5)^ Estudos em adultos mostraram que o TNG tópico aumenta o diâmetro interno das artérias periféricas, melhora a taxa de sucesso da canulação e reduz o risco de vasoespasmo e oclusões durante o cateterismo cardíaco.^(6-8)^

Nossa recente revisão sistemática^(9)^ encontrou apenas dois estudos que abordaram essa questão na população pediátrica. Um estudo utilizou um adesivo tópico de TNG,^(10)^ enquanto o outro estudo utilizou TNG subcutâneo.^(11)^ Ambos os estudos constataram que o TNG tópico melhorou as taxas de sucesso e reduziu o tempo necessário para a canulação arterial. No entanto, o tamanho da amostra era muito pequeno. Além disso, a população neonatal não estava representada nesses estudos.

Nossa hipótese é a de que a aplicação tópica de TNG na forma de um adesivo transdérmico proporcionaria a dilatação adequada da artéria radial e aumentaria a taxa de sucesso na primeira tentativa de canulação da artéria periférica em recém-nascidos.

Nosso objetivo é realizar um estudo-piloto controlado e randomizado para avaliar a eficácia e a segurança do TNG tópico no aumento do diâmetro da artéria radial em recém-nascidos.

## Métodos/desenho

### Objetivo e hipótese

Nosso objetivo é realizar um estudo controlado e randomizado para estabelecer a eficácia do TNG tópico no aumento do diâmetro da artéria radial em recém-nascidos.

Nossa hipótese é de que a aplicação tópica de TNG na forma de um adesivo transdérmico resultará em dilatação adequada (em pelo menos 25% do diâmetro basal) da artéria radial em recém-nascidos.

### Objetivos

Os objetivos específicos são:

1. Confirmar que a aplicação do adesivo de TNG por 30 a 60 minutos aumenta o diâmetro da artéria radial em recém-nascidos.
2. Confirmar que é seguro aplicar um adesivo de TNG por 30 a 60 minutos em recém-nascidos.
3. Estimar os valores médios das variações dentro do paciente no diâmetro da artéria periférica a partir dos valores basais, para informar a escolha da duração ideal da aplicação do adesivo de TNG no contexto da canulação em recém-nascidos.
4. Avaliar a viabilidade de um desenho de estudo futuro proposto para avaliar o impacto dos adesivos de TNG nas taxas de canulação na primeira tentativa em termos de recrutamento e coleta de dados.

#### Hipótese testada no estudo:

a aplicação tópica de TNG por 30 a 60 minutos aumenta o diâmetro da artéria radial em comparação com nenhuma aplicação.

### Desenho do estudo, cenário e período de tempo

Trata-se de estudo prospectivo, paralelo, controlado, randomizado e de centro único a ser conduzido na UTI neonatal do *Child and Adolescent Health Service* (CAHS) do *Perth Children's Hospital* (PCH), Perth, Austrália. A UTI neonatal do PCH é um centro de referência terciário com 30 leitos, que oferece cuidados intensivos e de alta dependência para recém-nascidos em estado grave. Mais de 800 lactentes são admitidos na UTI neonatal do PCH todos os anos, todos nascidos em outros hospitais. Esse estudo foi aprovado pelos *Human Research Ethics* (HREC) *and Governance Committees* do CAHS e encontra-se no *Australian New Zealand Clinical Trials Registry*. O estudo terá como objetivo recrutar pacientes candidatos (n = 60) de julho de 2023 a julho de 2024. Os pais ou responsáveis pelos recém-nascidos serão contatados e será obtido o Termo de Consentimento Livre e Esclarecido (TCLE). O plano de avaliação é mostrado na tabela 1. Este protocolo clínico foi preparado de acordo com a Declaração SPIRIT 2013.^(12)^

**Tabela 1:** Plano de avaliação

Termo de Consentimento Livre e Esclarecido
Revisão dos critérios de inclusão/exclusão
Randomização
Medição do diâmetro da artéria radial por ultrassom antes da intervenção por um operador cegado
Aplicação do adesivo de TNG/bandagem sem adesivo pela equipe de enfermagem não cegada (não envolvida no manejo diário do lactente) por 30 minutos, 45 minutos ou 60 minutos
Medição da pressão arterial realizada pelo profissional de enfermagem à beira do leito a cada 15 minutos até o fim da intervenção
Monitoramento da saturação de oxigênio e dos requisitos de FiO_2_ pelo profissional de enfermagem à beira do leito
Medição de gases sanguíneos pelo profissional de enfermagem à beira do leito após a intervenção para medir os níveis de metemoglobina
Remoção do adesivo de TNG/bandagem sem adesivo pela equipe de enfermagem não cegada (não envolvida no manejo diário do lactente) por 30 minutos, 45 minutos ou 60 minutos
Medição do diâmetro da artéria radial por ultrassom após a intervenção por um operador cegado

TNG - trinitrato de glicerilo; FiO_2_ - fração inspirada de oxigênio.

### Critérios de inclusão

1. Recém-nascidos com mais de 34 semanas de gestação, internados na UTI neonatal do PCH e para os quais é necessário um acesso arterial periférico para coleta de amostras ou monitoramento invasivo da pressão arterial.
2. Recém-nascidos com mais de 34 semanas de gestação internados na UTI neonatal, com cirurgia eletiva ou geral programada e para os quais seja necessária a canulação da artéria periférica para monitoramento hemodinâmico ou coleta frequente de sangue (perioperatório).

### Critérios de exclusão

1. Recém-nascidos submetidos a tentativas anteriores de canulação da artéria radial e aqueles com hematomas no local da canulação, resultados anormais do teste de Allen, estado de hipercoagulabilidade, coagulopatia e doença vascular periférica.
2. Recém-nascidos com sinais vitais instáveis, incluindo hipotensão, choque ou arritmias significativas.
3. Recém-nascidos que serão submetidos à cirurgia cardíaca.
4. Recém-nascidos com aumento da pressão intracraniana, hemorragia intracraniana e uso recente de sildenafila.
5. Recém-nascidos com uma deformidade visível na área da artéria radial.

## Intervenções

A TNG 5 mg (adesivo Transiderm-Nitro, 5mg/24 horas) será aplicada por 30 minutos, 45 minutos ou 60 minutos. Os recém-nascidos serão alocados no Grupo TNG ou no Grupo Controle. O TNG é um medicamento registrado no *Australian Therapeutic Goods Registry*. A *Therapeutic Goods Administration* (TGA) foi notificada sobre este estudo clínico (Número de referência: CT-2023-CTN-02133-1).

### Grupo TNG:

nos recém-nascidos deste grupo, será aplicado um adesivo de TNG (5mg) no local da canulação da artéria radial por 30 minutos (Grupo A, n = 15), 45 minutos (Grupo B, n = 15) ou 60 minutos (Grupo C, n = 15). Imediatamente após sua aplicação, o adesivo de TNG será coberto com uma bandagem adesiva Coban™ para assegurar o cegamento.

### Grupo Controle:

nos recém-nascidos desse grupo (Grupo D, n = 15), será aplicado um curativo adesivo Coban™ sem nenhuma intervenção na pele nua no local da artéria radial por 30 minutos (n = 5), 45 minutos (n = 5) ou 60 minutos (n = 5), para garantir o cegamento.

Apenas um local arterial por lactente será estudado para garantir que as amostras sejam independentes.

## Justificativa da dose da intervenção

Os pacientes randomizados nos braços da intervenção (Grupos A, B e C) receberão um adesivo de TNG 5mg (Transiderm-Nitro, 5mg/24 horas) aplicado por 30 minutos, 45 minutos ou 60 minutos, e sobre o adesivo será colocada uma bandagem adesiva Coban™. O Minitran 5 tem uma área de superfície de 6,7cm^2^ e a quantidade de TNG liberada em 24 horas é de 5mg. O sistema de liberação transdérmica Transiderm-Nitro foi projetado para fornecer liberação contínua e controlada de TNG através da pele intacta para superar os problemas de meia-vida curta e metabolismo de primeira passagem extensivo do TNG. A taxa de liberação do TNG é linearmente dependente da área do sistema aplicado.

A taxa de liberação do adesivo é de 0,2mg/hora. O início da ação do adesivo de TNG ocorre aos 30 minutos. Foi demonstrado que os níveis de TNG aumentam até atingir um estado estável dentro de 1 a 2 horas após a aplicação do adesivo de TNG, e os níveis diminuem com meia-vida de cerca de 30 minutos após a remoção do adesivo.^(13)^

Os estudos realizados por Hasanin et al. (adesivo transdérmico de TNG)^(10)^ e Jang et al. (injeção subcutânea de TNG) foram usados para dados farmacocinéticos e seleção de dose.^(11)^ Ambos os estudos usaram dose de 2,5 a 5mg/kg/hora. A maioria dos dados disponíveis sobre a dosagem de TNG foi derivada de estudos em adultos, não em pediatria.^(7,14,15)^ Nos estudos pediátricos, não foram relatados efeitos colaterais sistêmicos importantes, como hipotensão e metemoglobinemia.^(9)^

## Desfecho do estudo

O desfecho primário do estudo é o diâmetro da artéria radial. Após obter o TCLE dos pais do paciente, as imagens da artéria radial serão obtidas do pulso do lactente usando um aparelho de ultrassom portátil. O diâmetro da artéria radial será medido usando uma sonda linear de alta frequência (6 - 113MHz) com o sistema de ultrassom Philips EPIQ CVx Diagnostic Ultrasound.

As medidas serão obtidas no plano de eixo curto do antebraço distal, medialmente à borda do processo estiloide do rádio e no ponto com pulsação máxima da artéria radial, com o pulso em um ângulo de 45°.^(16)^ O ponto será marcado para orientar a intervenção e garantir a cobertura do local sobre a artéria periférica, que será medida após a intervenção. O operador será um neonatologista experiente, sem conhecimento do grupo de estudo. As medidas serão obtidas em ambas as artérias radiais no momento basal, antes da aplicação do adesivo de TNG/bandagem sem adesivo aos 30 minutos, 45 minutos ou 60 minutos após a aplicação.

## Critérios de retirada do participante

A pressão arterial média e sistólica será registrada a cada 15 minutos nos Grupos TNG e Controle até o fim da intervenção. Se algum participante do Grupo TNG ou Grupo Controle apresentar hipotensão (definida como uma queda > 30% na pressão arterial sistólica em relação aos valores basais) e/ou qualquer aumento repentino na necessidade de oxigênio com um aumento nos níveis de metemoglobina (determinado pela análise gasométrica), ele será retirado do estudo. Os participantes podem se retirar do estudo a qualquer momento.

## Desfechos

### Desfecho primário:

o desfecho primário será a variação no diâmetro da artéria radial pós-intervenção em relação aos valores basais. Esse desfecho será comparado entre os Grupos TNG e Controle.

### Desfechos secundários:

os desfechos secundários serão o diâmetro transversal da artéria radial pós-intervenção no membro ipsilateral e a variação percentual no diâmetro arterial radial no membro ipsilateral em relação aos valores basais. Também serão avaliadas as variações absolutas e percentuais pós-intervenção no diâmetro arterial radial do membro contralateral em relação aos valores basais.

### Desfechos de segurança:

neste estudo, serão avaliados os efeitos colaterais do TNG tópico, como hipotensão e metemoglobinemia.

## Cronograma do participante

O cronograma do estudo é mostrado na tabela 1.

Duração da participação do participante: 30 minutos a 1 hora.

## Tamanho da amostra

Pretendemos recrutar 60 pacientes (15 em cada braço: um controle e três braços TNG) ao longo de 12 meses, o que é viável, pois estimamos que aproximadamente 250 pacientes candidatos sejam admitidos na UTI neonatal a cada ano.

Justifica-se o tamanho da amostra com base no desejo de demonstrar que é possível obter uma vasodilatação razoável com a aplicação de adesivo de TNG em nossa população-alvo do estudo. Presumimos que as medidas do diâmetro da artéria radial nos participantes do estudo terão média de 0,76mm sem tratamento e desvio-padrão comum dentro do grupo de cerca de 0,18mm, conforme observado em um estudo de referência de recém-nascidos com idade entre zero e 6 meses.^(12)^ Com o tamanho de amostra de 15 para cada grupo (um grupo controle e três grupos com adesivo de TNG), a margem de erro em torno dos diâmetros médios populacionais estimados correspondentes é inferior a 1mm, e uma análise de variância unidirecional *omnibus* teria mais de 80% de poder para determinar se a aplicação do adesivo de TNG aumenta o diâmetro da artéria radial quando a média mínima do grupo tratamento difere da média do grupo controle em cerca de 0,19mm (alfa = 0,05). Espera-se que as análises baseadas na variação dos valores basais, que é o nosso desfecho primário, sejam mais poderosas do que as análises que avaliam os diâmetros de seção transversal, e a diferença detectável de seção transversal de 0,19mm corresponda a um aumento aproximado de 25% em relação ao valor estimado basal, o que é da ordem do observado anteriormente em crianças de zero a 2 anos que receberam injeções subcutâneas de TNG^(11)^ e substancialmente menor do que o observado após a aplicação do adesivo em crianças de 2 a 8 anos (aumento médio de 40% no diâmetro da artéria radial após 30 minutos e um aumento de 70% após 1 hora).^(10)^ Além disso, a potência será aumentada com a extensão da avaliação da vasodilatação induzida pelo TNG em uma análise de covariância que reduz a variação residual, ajustando os principais correlatos demográficos/clínicos do diâmetro arterial.

## Recrutamento

Os pais/responsáveis por um lactente selecionado serão contatados pelo investigador principal coordenador ou pelo representante dele e convidados a participar deste estudo. Os pais devem fornecer seu TCLE para a participação no estudo antes da realização de procedimentos cirúrgicos eletivos, para que a intervenção do estudo e a coleta de dados possam ser iniciadas.

## Distribuição das intervenções

### Geração de sequência aleatória

A alocação ao grupo será feita pelo farmacêutico de ensaios clínicos do PCH usando uma sequência de randomização gerada por computador para minimizar o viés de seleção.

### Ocultação da alocação

A ocultação será obtida por meio de envelopes opacos e lacrados. Após a obtenção do TCLE, um membro da equipe de enfermagem abrirá o próximo envelope lacrado opaco para alocar o lactente no grupo controle ou intervenção.

### Cegamento

As famílias, os profissionais de saúde e os pesquisadores do estudo serão cegados quanto à alocação ao grupo.

#### Não cegamento da equipe:

um membro da equipe de enfermagem que não esteja envolvido no manejo diário do recém-nascido será responsável por abrir o envelope, determinar a alocação ao grupo e aplicar o adesivo/placebo de TNG e remover o adesivo/placebo de TNG sem nenhuma outra participação no estudo.

#### Cegamento da equipe:

as medições da artéria radial por ultrassom serão realizadas por um operador que não terá conhecimento da intervenção. Além disso, o profissional de enfermagem à beira do leito que medir a pressão arterial a cada 15 minutos até o fim da intervenção também estará cegado.

Haverá um registro de cegamento e não cegamento da comissão de membros do estudo cegados e não cegados com suas funções listadas.

## Coleta, tratamento e análise de dados

### Métodos de coleta de dados

Serão coletados todos os dados clínicos, como as características do paciente, incluindo idade gestacional, idade cronológica, sexo, peso, localização, diagnóstico principal, sinais vitais, medicamentos e uso de ventilação (Tabela 2).

**Tabela 2:** Características dos pacientes e clínicas

		Grupo TNG n = 45	Grupo Controle n = 15
Peso ao nascer			
Idade gestacional			
Data de nascimento			
Sexo			
	Masculino		
	Feminino		
Idade na intervenção			
Diagnósticos			
	Perioperatório		
	Médico		
Medicamentos			
	Nenhum		
	Antibióticos		

TNG - trinitrato de glicerilo.

Após obter o TCLE dos pais de cada paciente, serão obtidas imagens da artéria radial do pulso do lactente usando um aparelho de ultrassom portátil. O diâmetro da artéria radial será medido com uma sonda linear de alta frequência tipo *hockey stick* (6 a 13MHz) com o sistema de ultrassom Philips EPIQ CVx Diagnostic Ultrasound.

O operador será um neonatologista experiente (membro da *Australian Society for Ultrasound in Medicine* e que realiza ultrassonografias rotineiramente na unidade) que não conhece o grupo de estudo. As medidas serão obtidas nas artérias radiais de ambos os membros no início do estudo, antes da aplicação do adesivo TNG/bandagem sem adesivo aos 30 minutos, 45 minutos ou 60 minutos após a aplicação.

### Tratamento de dados

O estudo envolve a coleta de dados clínicos e imagens da artéria radial por medição de ultrassom. Eles serão armazenados em um banco de dados protegido por senha no computador do hospital. O Setor de Tecnologia da Informação do Departamento de Saúde do CAHS estará envolvido para ajudar a proteger esses dados em um sistema protegido por senha. Não haverá compartilhamento ou reutilização de dados. Os registros serão armazenados em um armário fechado em um escritório protegido no Departamento de Neonatos do PCH. Os dados do estudo serão conservados conforme as diretrizes de saúde da Austrália Ocidental. Os dados coletados serão acessados apenas pelos membros autorizados da equipe do estudo, nomeados na solicitação do HREC do estudo.

### Métodos estatísticos

A tabela 3 apresenta uma descrição do cronograma e do método de avaliação dos parâmetros de eficácia e segurança. O operador e o examinador de ultrassom não terão conhecimento do estudo.

**Tabela 3:** Cronograma e método de avaliação dos parâmetros de eficácia e segurança

		Medido por	Análise
Desfecho primário de eficácia			
	Variação do diâmetro arterial radial ipsilateral pós-intervenção em relação aos valores basais	Ultrassom no local da canulação medido no momento da randomização do adesivo de trinitrato de glicerilo/placebo aos 30, 45 ou 60 minutos pós-intervenção	Análise de covariância
Desfechos secundários			
	Diâmetro transversal da artéria radial no membro ipsilateral	Medido pós-intervenção por ultrassom	Regressão linear
	Variação percentual em relação aos valores basais do diâmetro arterial radial no membro ipsilateral	Razão entre os diâmetros arteriais radiais pós-intervenção e basais medidos por ultrassom	Regressão linear
	Variação do diâmetro da artéria radial no membro contralateral (sem intervenção) em relação aos valores basais	Diferença e razão do diâmetro da artéria radial medido nos valores basais e pós-intervenção	Modelagem de regressão de efeitos mistos dos diâmetros da artéria radial em ambos os membros
Desfechos de segurança			
	Pressão arterial	Pressão arterial sistólica monitorada a cada 15 minutos	Modelagem de regressão de efeitos mistos das trajetórias dos pacientes
	Metemoglobinemia	Monitoramento da necessidade de oxigênio e uma análise de gasometria se aumentar a necessidade de oxigênio	Modelagem de regressão de efeitos mistos das trajetórias dos pacientes

### Monitoramento de dados e segurança

Criou-se o *Data and Safety Monitoring Committee* (DSMC) independente para garantir a segurança dos participantes do estudo e monitorar a qualidade do estudo e da coleta de dados. O DSMC tem um neonatologista e um farmacêutico sênior que são externos ao PCH e não estarão envolvidos no estudo. O DSMC revisará os dados do estudo e realizará análises provisórias após o recrutamento de 20 e 40 participantes para os principais desfechos de segurança: a incidência de hipotensão (definida como uma redução maior ou igual a 30% na pressão arterial sistólica em relação à leitura inicial) e metemoglobinemia (medida por análise de gases sanguíneos se algum lactente apresentar um aumento súbito na necessidade de oxigênio após a intervenção). Os termos do DSMC estão incluídos como um arquivo suplementar.

Todos os eventos adversos serão relatados ao HREC do CAHS e ao DSMC em 1 dia útil após o primeiro relato do evento.

## Ética e divulgação

Foi concedida aprovação ética para todos os aspectos deste estudo (RGS 0000005763). Quaisquer desvios deste protocolo somente ocorrerão com a aprovação prévia do HREC. O estudo será conduzido conforme os documentos orientadores de Boas Práticas Clínicas do Conselho Internacional de Harmonização e a *National Health and Medical Research Council National Statement* de conduta ética em pesquisa com seres humanos.

A intervenção proposta tem caráter não invasivo, e este será o primeiro estudo a avaliar o uso de TNG tópico para facilitar a canulação da artéria periférica em recém-nascidos. O adesivo de TNG está sendo usado em recém-nascidos para prevenir o vasoespasmo associado à isquemia local após a canulação da artéria periférica (em prematuros e a termo). Muitas UTIs neonatais em todo o mundo usam o TNG para essa finalidade. Portanto, não há implicações éticas para os recém-nascidos alocados no grupo intervenção.

Como recém-nascidos não podem dar consentimento, o TCLE será obtido prospectivamente dos pais/responsáveis pelos lactentes selecionados para a participação de seus recém-nascidos no estudo. Todos os esforços serão feitos para manter os mais altos padrões de assistência durante a obtenção do consentimento para a pesquisa clínica.

O investigador principal coordenador ou um representante fornecerá aos pais/responsáveis uma folha com informações sobre o teste. As informações do estudo serão discutidas em detalhes com os pais/responsáveis, e suas perguntas serão respondidas integralmente. Quando os pais/responsáveis estiverem satisfeitos com as informações e concordarem com a inscrição, eles assinarão o TCLE aprovado pelo HREC antes da randomização.

A participação neste estudo é voluntária. Os pais/responsáveis podem desistir da participação de seu lactente a qualquer momento, sem necessidade de justificar o motivo. Nenhuma informação adicional será coletada. Usaremos os dados e as imagens da artéria radial já coletados, a menos que os pais/responsáveis nos orientem a não fazê-lo. A desistência da participação não prejudicará quaisquer tratamentos médicos atuais ou futuros.

O TCLE original será arquivado no arquivo de dados do estudo, e os pais/responsáveis receberão uma cópia; será fornecida documentação sobre o tipo de estudo no qual o lactente está inscrito.

## DISCUSSÃO

Considerando a importância de se ter acesso arterial confiável em recém-nascidos e a dificuldade de canulação, qualquer intervenção que aumente as chances de sucesso do cateterismo arterial periférico ajudará a melhorar os desfechos clínicos em recém-nascidos em estado grave. Esse projeto de pesquisa ajudará a determinar se o uso tópico de TNG para canulação da artéria periférica é benéfico e se seus benefícios superam os possíveis efeitos colaterais. Este será o primeiro estudo a avaliar o uso de TNG tópico para facilitar a canulação da artéria periférica em recém-nascidos. Se nosso ECR piloto confirmar os benefícios do adesivo de TNG, ele preparará o caminho para ECRs mais abrangentes e multicêntricos que avaliem sua eficácia na melhora da taxa de sucesso na primeira tentativa de canulação da artéria periférica em recém-nascidos.
